# EANM perspective on clinical PET and SPECT imaging in schizophrenia-spectrum disorders: a systematic review of longitudinal studies

**DOI:** 10.1007/s00259-024-06987-1

**Published:** 2024-11-22

**Authors:** Antoine Rogeau, Anne Jetske Boer, Eric Guedj, Arianna Sala, Iris E. Sommer, Mattia Veronese, Monique van der Weijden-Germann, EANM Neuroimaging Committee, Donatienne Van Weehaeghe, Diego Cecchin, Antoine Verger, Nathalie L. Albert, Matthias Brendel, Igor Yakushev, Tatjana Traub-Weidinger, Henryk Barthel, Nelleke Tolboom, Francesco Fraioli

**Affiliations:** 1 Department of Nuclear Medicine, Lille University Hospital, Lille, France; 2 Department of Neuroscience, University Medical Center Groningen, University of Groningen, Groningen, the Netherlands; 3 Department of Nuclear Medicine, Aix Marseille Univ, APHM, CNRS, Centrale Marseille, Institut Fresnel, Hôpital de La Timone, CERIMED, Marseille, France; 4 Coma Science Group, GIGA-Consciousness, University Hospital of Liège, Liège, Belgium; 5 Department of Information Engineering, University of Padua, Padua, Italy; 6 Department of Neuroimaging, Institute of Psychiatry, Psychology and Neuroscience, King’s College London, London, UK; 7 Institute of Nuclear Medicine, University College London Hospitals NHS Foundation Trust, London, UK

**Keywords:** PET, SPECT, Psychosis, SSD, Schizophrenia, Molecular imaging, Nuclear medicine, Systematic review, Psychiatry

## Abstract

**Purpose:**

There is a need for biomarkers in psychiatry to improve diagnosis, prognosis and management, and with confirmed value in follow-up care. Radionuclide imaging, given its molecular imaging characteristics, is well-positioned for translation to the clinic. This systematic review lays the groundwork for integrating PET and SPECT imaging in the clinical management of schizophrenia-spectrum disorders.

**Methods:**

Systematic search of PubMed, Embase, Web of Science and Cochrane library databases was conducted from the earliest date available until February 2024. The focus was on longitudinal studies evaluating PET or SPECT imaging in individuals with a schizophrenia-spectrum or another psychotic disorders. Quality assessment was done using the Newcastle-Ottawa Scale (NOS), NIH scale for before-after studies and Cochrane Risk of Bias tool version 2 (Cochrane RoB2). Studies were further categorised into three groups: preclinical and diagnosis, predicting disease course or personalising treatment.

**Results:**

Fifty-six studies were included in the systematic review investigating in total 1329 patients over a median of 3 months. Over two-thirds used PET tracers, whereas the remaining studies employed SPECT tracers. The most frequently investigated system was dopaminergic transmission, followed by cerebral metabolism and blood flow. [^18^F]FDOPA demonstrated large effect size in predicting conversion of subjects at risk and treatment response. Additionally, treatment dosage could be optimised to reduce side effects using [^123^I]IBZM or [^11^C]raclopride.

**Conclusion:**

Molecular imaging holds significant promise for real-life application in schizophrenia, with two particularly encouraging avenues being the prediction of conversion/response to antipsychotic medication and the improved management of antipsychotic dosage. Further longitudinal studies and clinical trials will be essential for validating both the clinical effectiveness and economic sustainability, as well as for exploring new applications.

**Supplementary Information:**

The online version contains supplementary material available at 10.1007/s00259-024-06987-1.

## Introduction

The World Health Organization (WHO) ranks mental disorders among the top 10 global causes of years lived with disability (YLD) [1]. Schizophrenia-spectrum disorders (SSD), characterised by an impaired perception of reality, stand out with a high societal cost and twice the mortality rate of the general population [2, 3]. Although it has long been shown that these conditions have a brain-basis, albeit with modest deviations, biomarkers are still awaited for improved management of patients.

Radionuclide imaging, with its ability to visualise subtle molecular interactions, is well-positioned to uncover the underlying neural mechanisms of psychotic symptoms [4]. Research employing single photon emission computed tomography (SPECT) and positron emission tomography (PET) has already contributed to advancing our understanding [5], and this growing knowledge could transform how psychotic disorders are managed.

Current thinking suggests that increased presynaptic dopamine synthesis may underly positive symptoms in at least a subset of patients with schizophrenia, presenting an opportunity for early diagnosis and treatment strategies [6, 7]. However, there is presently no standardised application defined in psychiatry for tools able to investigate neuronal function. Imaging techniques, such as [^18^F]FDG PET or DATscan/[18F]FDOPA PET, are primarily used to exclude psychiatric or iatrogenic causes in patients with neurological symptoms [8, 9]. This reiterates that although an improved understanding of disorders is helpful in refining the nosography, the transition to clinical application will not happen automatically.

Longitudinal studies will be crucial in bridging this gap, as they can identify and track key events over time, establish causal relationships, and confirm outcomes upon follow-up [10]. For example, they were instrumental in clarifying the role of dopamine in the development of schizophrenia [11]. Given the chronic nature of many SSD, such study set-ups will be best suited in optimising the use of molecular imaging at different stages of the disease – ranging from preclinical phase to diagnosis, treatment, remission and relapse prediction.

In the current article, we present the first systematic review of longitudinal studies utilising SPECT or PET imaging in SSD. While previous reviews have focused on specific neurotransmitters [12, 13], a systematic review from an imaging perspective to guide physicians on potential future indications has never been presented. Therefore, the aim of this paper is to critically assess the evidence that supports the role of radionuclide neuroimaging in the clinical routine management of patients with schizophrenia.

## Methods

The systematic review is reported according to a predefined internal protocol and written according to the Preferred Reporting Items for a Systematic Review and Meta-Analysis (PRISMA) statement [14]. The PRISMA checklist can be found in the supplementary materials (Supplementary Table 1). The complete study protocol can also be found in the supplementary materials. No ethical approval or informed consent was required.

### Search strategy

The search strategy followed a serial approach to identify studies for inclusion in this review. The first step was to identify pivotal studies by entering various combinations of the following terms in PubMed: “psychosis”, “SSD”, “schizophrenia”, “PET/CT”, “PET/MRI”, “PET”, “SPECT”, “SPECT/CT”, “longitudinal studies” and “follow-up”. Following this, Medical Subject Headings (MeSH) terms were extracted from these studies following the Population, Intervention and Context (PICo) framework. In our case, population represented SSD, intervention imaging and context longitudinal follow-up. The second step was to exhaustively search the PubMed, Embase, CENTRAL and Web of Science databases using the selected MeSH terms (Supplementary Table 2). The search process was concluded on February 14th, 2024. Finally, all results were extracted and imported into Rayyan (https://www.rayyan.ai) and duplicates were removed.

## Study selection

Two authors (AR and AJB) independently reviewed studies in Rayyan to assess for inclusion in the review. Inclusion criteria were (1) studies performed in humans with psychosis, schizophrenia or at clinical high risk of psychosis, (2) using SPECT or PET imaging, (3) with a longitudinal design defined as following subjects at minimum two timepoints ≥ 7 days, (4) and original studies. Exclusion criteria were (1) studies done in healthy human, animal or in vitro models, (2) clinical trials investigating non-approved drugs, (3) case reports or small series of cases (≤ 5 subjects), (4) letters to editors or commentaries, (5) abstracts presented at conferences with no full text, (6) phantom studies, (7) previous reviews and meta-analyses. No additional exclusion criteria were applied. There was no language restriction.

Both researchers independently screened and critically assessed each study for relevance based on title and abstract. After this screening step, final selection of articles was done using full texts. If full text articles were not retrievable, authors were contacted to ascertain whether a full text article was published or obtainable. Disagreements were resolved by reviewing discrepancies until agreement was reached and asking a third opinion (FF) if agreement could not be reached.

## Data extraction

Data extraction was performed independently by two authors (AR and FF). For each article, they collected information on the tracer used, system investigated (e.g., cerebral perfusion, striatal dopamine receptors), type of camera (SPECT, SPECT/CT, PET, PET/CT, PET/MRI), attenuation correction, number of subjects, diagnosis, and follow-up period. Detailed information on imaging protocols in studies using PET tracers with recent EANM recommendations was also collected [8, 9]. When feasible, an effect size measure, such as Cohen’s d, was extracted. If the studies varied significantly in how data was presented, making effect size computation impractical, p-values were collected instead. Both authors then reviewed and compiled the data collaboratively.

## Quality assessment

Quality assessment of the selected studies was done in consensual agreement by two authors (AR and FF). Longitudinal studies varying in their design (randomised or non-randomised, with or without control group), adapted tools for quality assessment were used following recommendations [15]. Non-randomised studies including a control group (“case control” studies) were assessed using the Newcastle-Ottawa scale (NOS) [16]. Non-randomised studies with no control group (“before after” studies) were assessed using the National Institute of Health (NIH) tool for pre-post studies with no control group (https://www.nhlbi.nih.gov/health-topics/study-quality-assessment-tools). Finally, studies employing a randomised controlled design (“randomised” studies) were evaluated using the Cochrane Risk of Bias 2 (RoB2) [17]. Each study was rated as having ‘Poor’, ‘Fair’ or ‘Good’ quality based on these criteria.

### Data analysis

We investigated each tracer used in each study and what systems they investigate. Data are presented for each system tracer. In addition, studies were classified into 3 categories depending on where they are most susceptible to transfer into clinical practice (preclinical and diagnostic stage, predicting disease course and personalising treatment). Results are presented following this framework. Considering heterogeneity of the studies, a meta-analysis was not performed. Detail of each study with main findings are summarised (Table 1). Most investigated variables across studies are presented using bubble and forest plots.

**Table 1:** Summary of studies

Articles	Tracer	Camera	Target	Design	Subjects	Follow-up	Findings
Assess risk and help diagnosis							
Allen et al. (2012) [18]	[^18^F]FDOPA	PET	Brainstem DSC	Case control	21 UHR − 5 UHR-t − 16 UHR-nt 14 HC	24.7 months	Elevated brainstem Ki^cer^ in UHR-t vs. UHR-nt (*p* < .01) and trend in UHR-t vs. HC (*p* = .10).
Corripio et al. (2006) [19]	[^123^I]IBZM	SPECT	Striatal DRA	Case control	18 FEP − 11 SZ − 7 non-SZ 18 HC	24 months	Elevated baseline striatal/occipital ratio in subjects who were later diagnosed SZ vs. non-SZ (*p* = .0005), and high diagnostic probability (AUC = 0.96).
Corripio et al. (2011) [20]	[^123^I]IBZM	SPECT	Striatal DRA	Case control	37 FEP − 25 SZ − 12 non-SZ 18 HC	12 months	Elevated baseline striatal/frontal ratio in subjects who were later diagnosed SZ vs. non-SZ (*p* < .001, AUC = 0.8), and no difference between non-SZ and HC (*p* = .9).
Howes et al. (2011) [11]	[^18^F]FDOPA	PET	Striatal DSC	Case control	24 UHR − 9 UHR-t − 15 UHR-nt 29 HC	36 months	Elevated whole striatum and associative Kicer in UHR-t vs. HC (*p* = .004, Cohen’s d = 1.18; *p* = .015, Cohen’s d = 1.24) and in UHR-t vs. UHR-nt (*p* = .036; *p* = .015).
Howes et al. (2020) [21]	[^18^F]FDOPA	PET/CT	Striatal DSC	Case control	35 UHR − 10 UHR-t − 25 UHR-nt 19 HC	15 months	Correlation between baseline striatal Ki^cer^ and worsening of positive symptoms (R^2^ = 0.12, *p* < .05). No significant difference in baseline Ki^cer^ between UHR-t and UHR (*p* = .28).
Mané et al. (2011) [22]	[^123^I]FP-CIT	SPECT	Striatal DAT	Case control	14 FEP − 12 SZ − 2 schizoaffective 7 HC	49.8 months	No significant changes in striatal/occipital ratio between patients and HC (*p* = .49). Negative correlations between baseline and difference in striatal/occipital ratio (*p* = .035 and 0.033).
Modinos et al. (2021) [23]	[^18^F]FDOPA	PET/CT & PET	Striatal DSC	Case control	50 UHR − 25 good outcome − 25 poor outcome 28 HC	14.8 months	Negative association between right hippocampus rCBF and Ki^cer^ in UHR-poor outcome (p_fwe_ = 0.012), which correlated with worsening of positive symptoms (*p* = .041).
Molina et al. (2005) [24]	[^18^F]FDG	PET	Frontal and occipital metabolism	Case control	13 FEP − 6 SZ − 7 non-SZ 8 HC	24 months	Reduced bilateral frontal metabolism in SZ vs. non-SZ (left, *p* = .03 and right, *p* = .01) and on the left in SZ vs. HC (*p* = .01). No difference in the occipital cortex (*p* = .78).
Molina et al. (2005) [25]	[^18^F]FDG	PET	Cerebral metabolism	Case control	21 FEP − 11 SZ − 10 non-SZ 16 HC	24 months	Lower prefrontal and higher hippocampal metabolism in SZ vs. HC (*p* = .09 and 0.031). No difference between non-SZ and HC (*p* > .05).
Predict course of disease							
Andersen et al. (2020) [26]	[^123^I]IBZM	SPECT/CT	Striatal DRA	Case control	21 FEP 23 HC	6 weeks	Significant reduction in symptom severity at 44.65% average occupancy (*p* < .001). No correlation between symptoms and receptor occupancy (*p* > .05).
Brewer et al. (2007) [27]	[^15^O]H_2_O	PET	Cerebral blood flow during Stroop task	Case control	8 FEP − 8 SZ 8 HC	8 weeks	Greater recruitment of frontal regions in FEP (*p* < .0001) vs. greater recruitment of posterior regions in HC (*p* < .0001) at follow-up compared to baseline.
Corson et al. (2002) [28]	PET blood flow tracer (assumed to be [^15^O]H_2_O)	PET	Subcortical blood flow before/after antipsychotics	Case control	13 SSD	27 days	Elevated rCBF to both caudate and putamen at follow-up compared to baseline (*p* = .009 and 0.041, respectively).
De Picker et al. (2019) [29]	[^18^F]PBR111	PET/CT	Cerebral TSPO binding before/after antipsychotics	Case control	10 SSD 16 HC	8 weeks	Three-way interaction between time of scan, age and cohort mediated TSPO V_T_ (*p* = .020). Different V_T_ patterns between SSD and HC.
Eisenberg et al. (2017) [30]	[^15^O]H_2_O & [^18^F]FDOPA	PET	Striatal blood flow before/after antipsychotics & DSC before antipsychotics	Randomised controlled	30 SZ had [15O]-water − 18 SZ had [18 F]-FDOPA	3 weeks	Elevated rCBF on antipsychotic compared to placebo (*p* < .05). Excited PANSS score change predicted rCBF change (*p* < .05). Ventral striatal Ki^cer^ predicted ventral striatal rCBF change (*p* = .012).
Erkwoh et al. (1997) [31]	[^99m^Tc]HMPAO	SPECT	Cerebral blood flow before/after antipsychotics	Case control	22 FEP − 22 SZ 20 HC	Until remission (duration not mentioned)	No reduction was observed in rCBF between before and after treatment (*p* > .05).
Gur et al. (1995) [32]	[^18^F]FDG	PET	Cerebral metabolism	Case control	42 SZ − 22 FEP − 20 PT 42 HC	24 months	Higher metabolism (e.g. lateral lenticular, *p* = .004), and lower relative left-to-right hemispheric values associated with better outcome.
Jauhar et al. (2019) [33]	[^18^F]FDOPA	PET/CT	Striatal DSC	Case control	20 FEP − 8 BD − 12 SZ	6 months	Correlation between baseline associative Ki^cer^ and change in total/positive symptoms (*p* = .045 and 0.03). No change in Ki^cer^ before and after treatment (*p* = .47).
Jauhar et al. (2019) [34]	[^18^F]FDOPA	PET/CT	Striatal DSC	Case control	26 FEP 14 HC	4 weeks	Elevated associative Ki^cer^ in responders vs. non-responders and HC (*p* < .01, Cohen’s d = 1.55 and 1.31). Correlation with improvement in symptoms (positive symptoms, *p* < .001).
Jauhar et al. (2023) [35]	[^18^F]FDOPA	PET/CT	Striatal DSC	Case control	18 FEP 20 HC	4 weeks	Negative relationship between Ki^cer^ and anterior cingulate glutamate at baseline that disappeared after antipsychotic (*p* = .018).
Kim et al. (2021) [36]	[^18^F]FDOPA & [^11^C]raclopride	PET/CT	Striatal DSC & DRA before/after stopping antipsychotic	Case control	25 FEP − 10 relapse − 15 no relapse 14 HC	6 weeks	Correlation between baseline Ki^cer^ and time to relapse (*p* = .018) in relapsed subjects. No difference in BPND (*p* = .261).
Laurikainen et al. (2020) [37]	[^11^C]PBR28	PET/CT	Cerebral TSPO binding	Case control	14 FEP 15 HC	12 months	Lower V_T_ in FEP vs. HC (*p* = .026, Cohen’s d = 0.94). No association with symptom change (*p* > .05).
Livingston et al. (1998) [38]	[^99m^Tc]HMPAO	SPECT	Cerebral blood flow before/after antipsychotics	Case control	27 SZ 38 HC	6 months	Trend of decreased frontal metabolism in SZ vs. HC at baseline (*p* = .054). Increased putamen metabolism at follow-up compared to baseline (*p* < .004).
Lubeiro et al. (2016) [39]	[^18^F]FDG	PET	Cerebral metabolism	Case control	121 SZ − 64 FEP 60 HC 22 BD	6 months	Subjects that showed decreased left caudate/thalamus metabolism at baseline (*p* < .004) also showed a worsening trend of negative symptoms (*p* = .08).
Molina et al. (2003) [40]	[^18^F]FDG	PET	Cerebral metabolism before/after antipsychotics	Case control	45 SZ − 11 FEP − 34 PT	6 months	Elevated motor area metabolism in PT vs. treatment-naive FEP (p_fwe_ < 0.05). Uncorrected elevated metabolism in the primary visual area and insula after treatment (p_uncorr_ < 0.001).
Nørbak-Emig et al. (2017) [41]	[^123^I]epidepride	SPECT	Extrastriatal DRA before/after antipsychotics	Randomised controlled	20 FEP − 20 SZ 19 HC	3 months	No correlation between BPND and regional grey matter volume at baseline or follow-up (*p* > .3).
Novak et al. (2005) [42]	[^99m^Tc]ECD	SPECT	Cerebral blood flow before/after antipsychotics	Before-after	7 FEP − 7 SZ	9 weeks	Increased perfusion in frontal regions before and after treatment (*p* < .05).
Park et al. (2019) [43]	[^18^F]FDG	PET/CT	Cerebral metabolism before/after treatment	Case control	16 SZ − 8 aripiprazole + amisulpride − 8 aripiprazole + CBT 15 HC	12 weeks	Increased frontal and occipital metabolism in all SZ after treatment (p_fdr_ < 0.04). Uncorrected increase of frontal metabolism in amisulpride vs. CBT (p_uncorr_ < 0.001).
Sigvard et al. (2022) [44]	[^18^F]FDOPA	PET/CT	Striatal DSC	Case control	15 FEP 31 HC	6 months	Correlation between decarboxylation rate (k_3_) and baseline positive symptoms (*p* < .001) as well as improvement at follow-up (*p* = .006).
Szymanski et al. (1996) [45]	[^18^F]FDG	PET	Cerebral metabolism	Case control	16 SZ − 8 with tardive dyskinesia − 8 without	36 months	Elevated temporolimbic, brainstem and cerebellar metabolism along reduced parietal/cingulate metabolism at baseline in subjects who developed dyskinesia (*p* < .05).
Wong et al. (2022) [46]	[^18^F]FDOPA	PET/MRI	Striatal DSC	Case control	19 FEP − 9 DD − 10 SZ	3 months	Association between Ki^cer^ at baseline and fewer negative symptoms at follow-up in SZ (*p* = .01) while no association in DD (*p* = .64).
Wulff et al. (2020) [47]	[^123^I]IBZM	SPECT/CT	Striatal DRA	Case control	22 FEP − 22 SZ 23 HC	6 weeks	Correlation between improvement of fMRI signal to reward task and receptor occupancy (*p* = .035) in treatment responders.
Personalise treatment management							
Agid et al. (2007) [48]	[^11^C]raclopride & [^11^C]FLB-457	PET	Striatal and extrastriatal DRA before/after antipsychotics	Randomised controlled	14 FEP − 5 low-dose risperidone or olanzapine − 9 high-dose	15 days	Striatal occupancy predicted response for positive (*p* = .01) but not negative (*p* = .5) symptoms. Extrastriatal occupancy did not predict response (*p* > .05).
Bernardo et al. (2001) [49]	[^123^I]IBZM	SPECT	Striatal DRA before/after antipsychotics	Randomised controlled	27 SZ − 13 haloperidol − 14 olanzapine	4 weeks	No relationship between occupancy and symptoms (*p* > .05). Correlation between occupancy and EPS (*p* = .01).
de Haan et al. (2003) [50]	[^123^I]IBZM	SPECT	Striatal DRA	Randomised controlled	20 SZ − 9 olanzapine − 11 haloperidol	6 weeks	Occupancy between 60–70% associated with optimal subjective well-being (*p* = .004).
Fervaha et al. (2016) [51]	[^11^C]raclopride	PET	Striatal DRA before/after dose reduction	Before-after	38 SZ	3 months	No relationship between occupancy and negative symptoms at baseline or follow-up (*p* > .05).
Graff-Guerrero et al. (2015) [52]	[^11^C]raclopride	PET	Striatal DRA before/after dose reduction	Before-after	33 late-life SZ − 10 EPS − 23 non-EPS	3 months	No occupancy difference in EPS and non-EPS (*p* > .05) but more likely to occur with occupancy > 60%. Lowest occupancy with clinical stability was 50%.
Iwata et al. (2016) [53]	[^11^C]raclopride	PET	Striatal DRA before/after dose reduction	Before-after	38 SZ	3 months	Association between prolactin and occupancy (*p* < .04). Occupancy ≥ 66% more likely to have hyperprolactinemia (*p* = .03).
Kapur et al. (1996) [54]	[^11^C]raclopride	PET	Striatal DRA before/after antipsychotics	Before-after	7 SZ	2 weeks	Occupancy ranging from 53–74% (mean = 67%, SD = 7%) under 2 mg/day haloperidol.
Kapur et al. (1998) [55]	[^11^C]raclopride & [^18^F]setoperone	PET	Striatal DRA and prefrontal SRA before/after olanzapine	Randomised controlled	12 SZ	8 weeks	Saturation of 5-HT2 receptors at any dose. Hyperbolic relationship between dopamine receptor occupancy and dose (p not reported).
Kapur et al. (2000) [56]	[^11^C]raclopride & [^18^F]setoperone	PET	Striatal DRA and prefrontal SRA before/after quetiapine	Randomised controlled	12 SZ	12 weeks	Greater effect 5-HT2 than dopamine receptor occupancy (*p* < .001). Correlation between dose and 5-HT2 occupancy (*p* < .001).
Mishra et al. (2022) [57]	[^99m^Tc]ECD	SPECT/CT	Cerebral blood flow before/after treatment	Randomised controlled	60 treatment-resistant SZ − 30 ECT − 30 clozapine	24 weeks	Higher prefrontal and temporal perfusion at follow-up in ECT vs. clozapine (*p* < .05). Correlation between changes in positive symptoms and increased left temporal perfusion in ECT (*p* = .017).
Mizrahi et al. (2011) [58]	[^11^C]PHNO	PET	Subcortical DRA before/after antipsychotics	Before-after	8 FEP − 6 SZ − 2 schizoaffective	2.5 weeks	Lower BPND in globus pallidus and substantia nigra at follow-up compared to baseline suggesting possible upregulation.
Moresco et al. (2004) [59]	[^18^F]fluoro-ethyl-spiperone	PET	Cerebral D/SRA before/after antipsychotics	Randomised controlled	15 treatment-resistant SZ − 9 olanzapine − 6 clozapine	8 weeks	No binding difference in the cortex reflecting 5-HT2 occupancy (*p* > .05) while higher striatal dopamine receptor occupancy in olanzapine vs. clozapine (*p* < .005).
Nakajima et al. (2016) [60]	[^11^C]raclopride	PET	Striatal DRA before/after dose reduction	Before-after	32 SZ − 20 olanzapine − 12 risperidone	12 weeks	Correlation between occupancy and plasma levels following a hyperbole saturation model (olanzapine *p* < .001, risperidone *p* < .002).
Nørbak-Emig et al. (2016) [61]	[^123^I]epidepride	SPECT	Frontal DRA before/after antipsychotics	Randomised controlled	22 FEP SZ − 13 risperidone − 9 zuclopenthixol	3 months	Correlation between occupancy and slower executive functions (*p* = .003 and 0.048). Correlation between baseline BPND and positive symptom reduction (*p* < .016).
Pavics et al. (2004) [62]	[^123^I]IBZM	SPECT	Striatal DRA on quetiapine high-dose and lower dose	Before-after	10 SZ	12 months	Occupancy changes correlated with time until relapse (*p* < .01). Striatum/occipital ratio at baseline higher in patients with relapse (*p* < .01).
Pickar et al. (1996) [63]	[^123^I]IBZM	SPECT	Striatal DRA before/after clozapine dose reduction	Before-after	13 SZ	3 weeks	No correlation between lesser occupancy and symptom worsening (*p* > .05). Correlation between plasma levels and occupancy (*p* = .003).
Pilowski et al. (1996) [64]	[^123^I]IBZM	SPECT	Striatal DRA	Case control	32 SZ − 6 olanzapine − 10 clozapine − 10 typical antipsychotics − 6 risperidone	4 weeks	Lesser occupancy in olanzapine vs. typical antipsychotics (*p* = .03), risperidone (*p* = .025) and no difference vs. clozapine (*p* > .05) while no difference in symptom improvement (*p* > .05).
Potkin et al. (2014) [65]	[^18^F]fallypride	PET	Basal ganglia DRA before/after lurasidone	Randomised controlled	10 SZ 7 schizoaffective	> 1 week (not precisely mentioned)	Correlation between plasma concentration and occupancy (*p* < .0006). Correlation between occupancy positive symptom (*p* < .011).
Rajji et al. (2017) [66]	[^11^C]raclopride	PET	Striatal DRA before/after dose reduction	Before-after	31 late-life SZ 6 late-life schizoaffective	2 weeks	Relationship between BPND and cognition at follow-up (*p* = .005) not seen at baseline.
Rasmussen et al. (2011) [67]	[^18^F]altanserin	PET	Cerebral SRA before/after quetiapine	Before-after	15 FEP − 15 SZ	6 months	Hyperbolic relationship between occupancy and positive symptom changes (*p* < .001). Optimal occupancy level between 60–70%.
Rasmussen et al. (2014) [68]	[^18^F]altanserin	PET	Neocortical SRA before/after quetiapine	Before-after	15 FEP − 15 SZ	6 months	Correlation between BMI increase and BPND at baseline (*p* = .022) as well as occupancy at follow-up (*p* = .038).
Schröder et al. (1998) [69]	[^123^I]IBZM	SPECT	Striatal DRA before/after antipsychotics	Before-after	15 SZ	28 days	Correlation between basal ganglia/frontal ratio at baseline and EPS (*p* < .05). Trend of decreased ratio before/after treatment in good outcome subjects while increased in poor outcome (*p* = .06).
Tauscher-Wisniewski et al. (2002) [70]	[^11^C]raclopride	PET	Striatal DRA before/after quetiapine	Before-after	14 FEP − 14 SSD	12 weeks	Correlation between plasma levels and peak occupancy (0.003). Mean peak occupancy of 62% ± 10% and mean trough occupancy 14% ± 8%.
Uchida et al. (2012) [71]	[^11^C]raclopride	PET	Striatal DRA before/after dose reduction	Before-after	9 late-life SZ	3 months	EPS associated to occupancy > 70% and improved upon dose reduction. Clinical worsening seen for occupancy ≤ 52%.
Wulff et al. (2015) [72]	[^123^I]IBZM	SPECT/CT	Striatal DRA	Case control	24 FEP − 24 SZ 26 HC	6 weeks	Correlation between lower BPND at baseline and improvement of total/positive symptoms (*p* = .003 and 0.048) but not negative (*p* = .33). Correlation between higher occupancy and lower functioning at follow-up (0.049).

*BD *bipolar disorder, *BPND *nondisplaceable binding potential, *CBT *cognitive behavioural therapy, *DAT *dopamine autotransporter, *DD *delusional disorder, *DSC *dopamine synthesis capacity, *DRA *dopamine receptor availability, *ECT *electroconvulsive therapy, *FEP *first-episode psychosis, *fMRI *functional magnetic resonance imaging, *HC *healthy controls, *Ki*^cer^ influx rate constant of [18F]–FDOPA, *p*_fdr_ false-discovery rate p, *p*_fwe_ familywise error p, *PT* previously treated, *rCBF *regional cerebral blood flow, *SRA *serotonin receptor availability, *SSD *schizophrenia-spectrum disorders, *SZ *schizophrenia, *UHR *ultra-high risk, *UHR-t *transitioned ultra-high risk, *UHR-nt *non-transitioned ultra-high risk

## Results

### Eligible studies

**Fig. 1:**
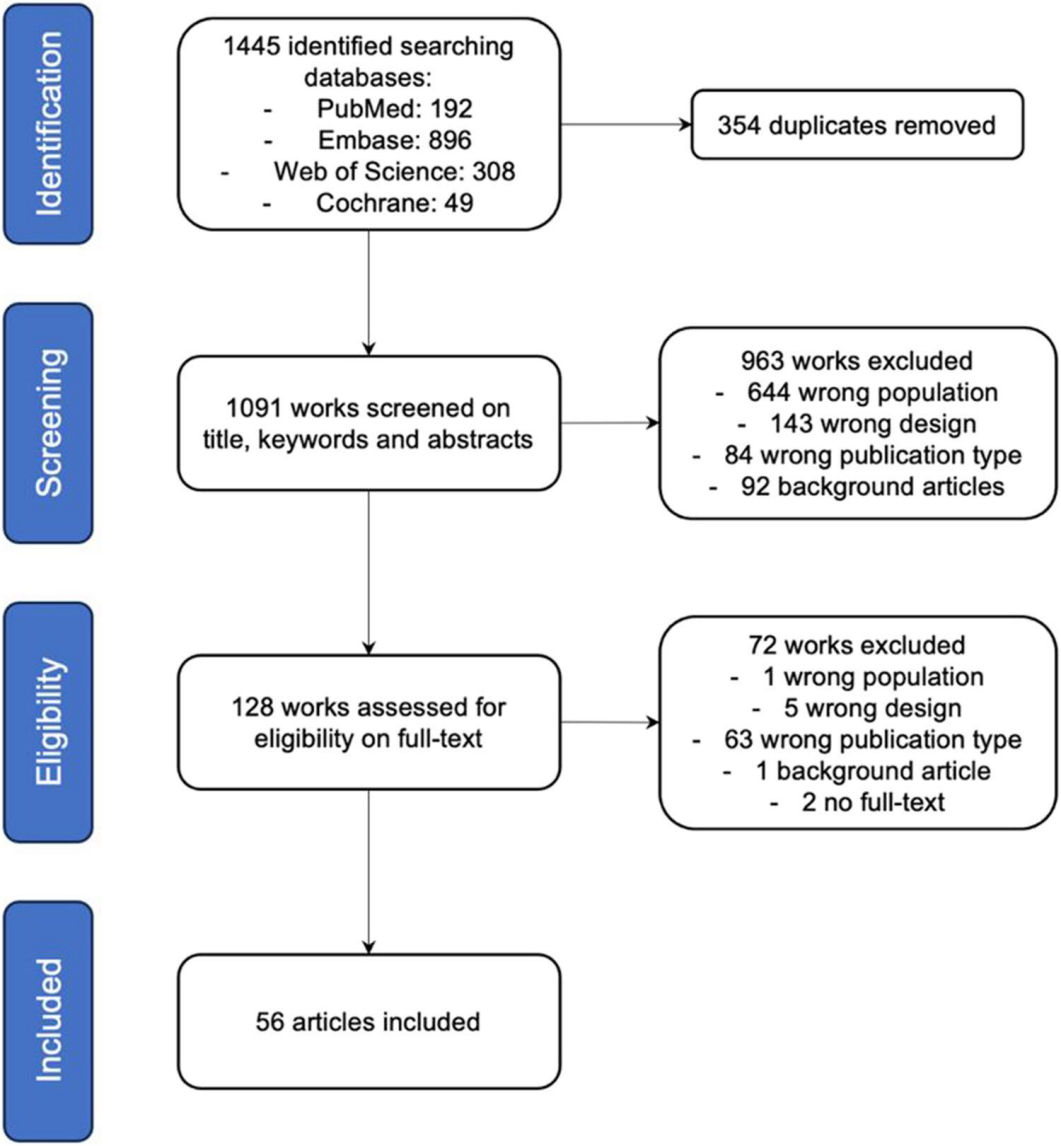
PRISMA flow chart of selected studies. Wrong population: studies not using psychotic human subjects, wrong design: studies not using PET/SPECT and a longitudinal design, wrong publication type: studies with no full text or no original data, background articles: previous reviews or opinion papers

The systematic search yielded 1445 studies, of which 354 duplicates were removed (Fig. 1). A total of 963 works were excluded on the basis of title, keywords and abstracts. These consisted in 644 studies which were not done in human psychotic subjects, 143 studies with wrong design, mostly not incorporating PET or SPECT, 84 wrong publication types including case reports, comments and editorial and 92 background articles, mostly reviews. Kappa agreement between both reviewers (AR and AJB) was excellent at 95.4%. Full-text was queried for 128 studies and 72 of them were subsequently excluded, mostly conference abstracts with no full-text. All included studies were in English. Included studies were [11, 18–72].

## Quality assessment

**Fig. 2:**
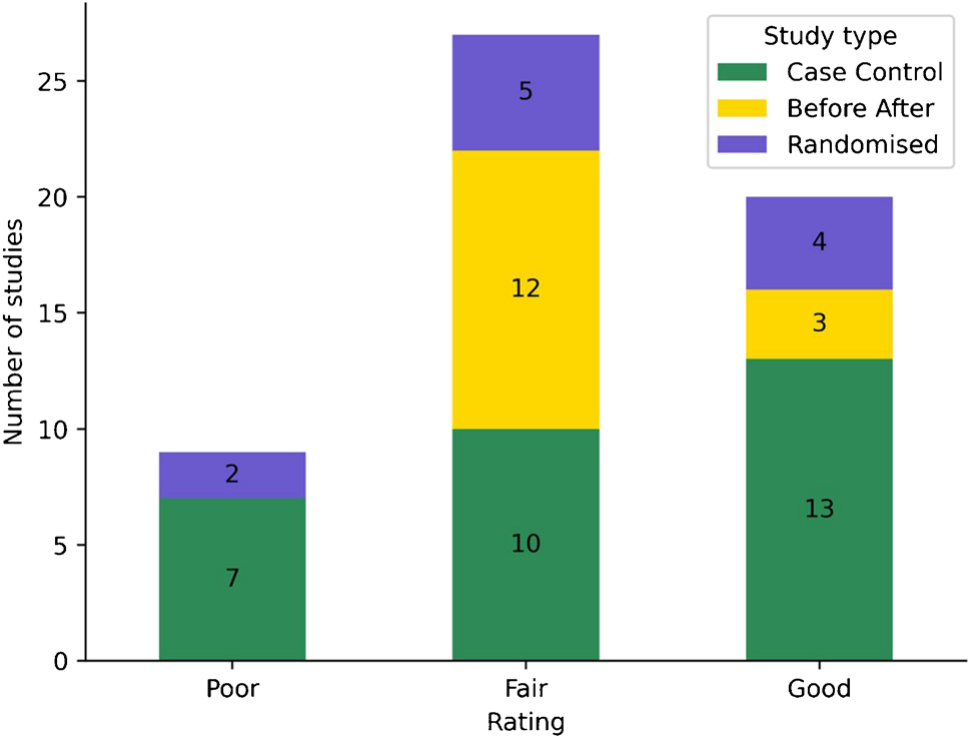
Quality assessment of the different study types. X-axis represents quality rating. Y-axis represents number of studies

Detailed quality assessment for each study can be found in Supplementary Tables 3, 4 and 5. The majority of studies demonstrated good or fair quality (35.7% and 48.2%, respectively). There was balanced distribution of case control and randomised studies across the 3 categories while most of before-after studies had fair quality and none of them had poor quality (Fig. 2).

### Technical characteristics

**Fig. 3:**
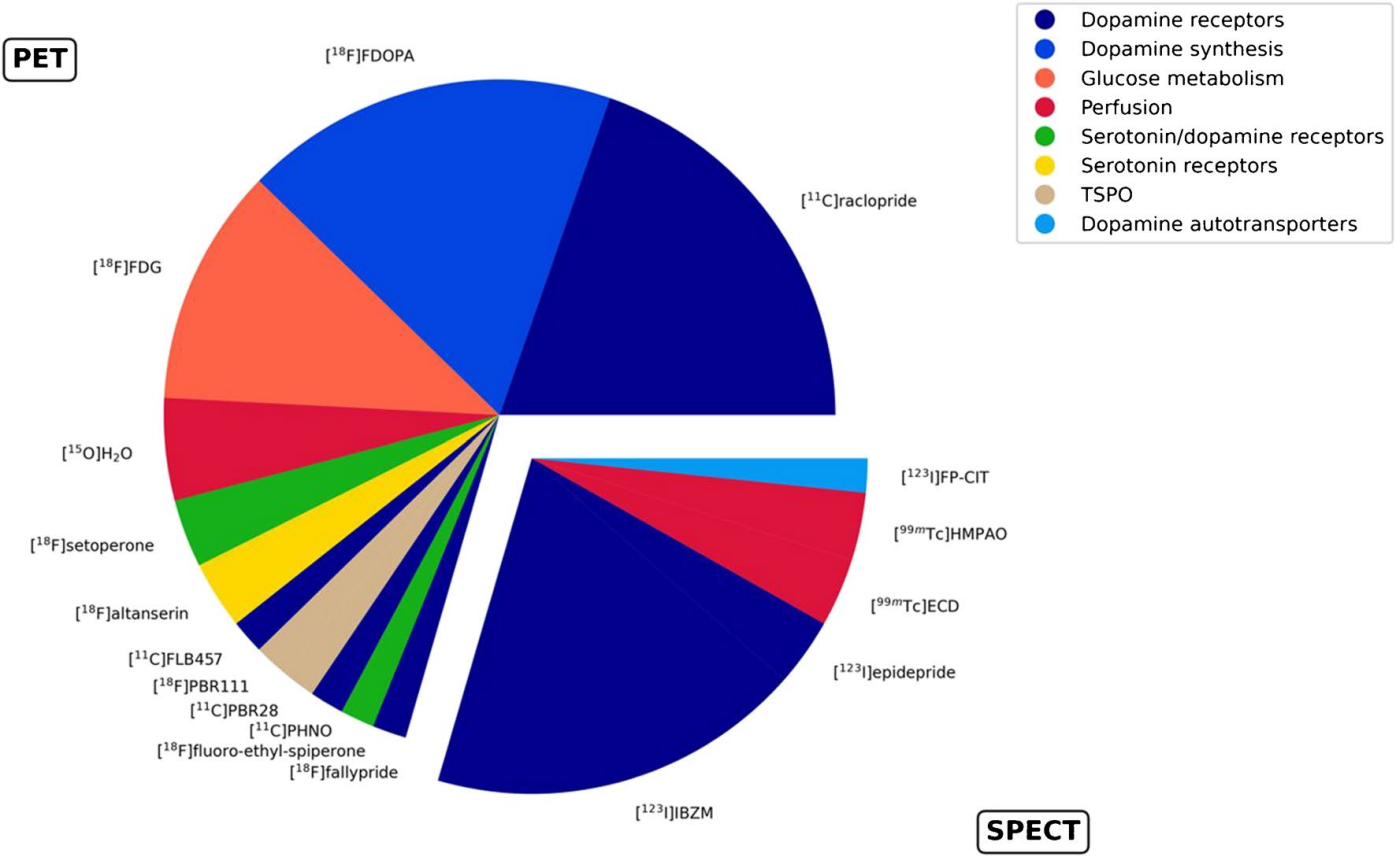
Overview of studies. PET, positron emission tomography; SPECT, single-photon emission computed tomography; TSPO, translocator protein

A total of 1329 patients with an SSD, another psychotic disorder or at ultra-high risk of developing psychosis (UHR) were investigated. Follow-up duration extended from 2 weeks to over 4 years. Median follow-up duration was 91.3 days (3 months). Thirty-eight studies (67.9%) used PET and 18 (32.1%) SPECT imaging (Fig. 3). Five studies included two tracers, and others used one tracer. Most studies (69.6%) investigated the dopamine system at different stages (presynaptic synthesis or transporter/receptor availability). The second most prevalent facet was glucose metabolism and cerebral blood flow (both accounting for 12.5% of studies). Reflecting this, the most common tracer was [^11^C]raclopride (19.7%), followed by [^123^I]iodobenzamide ([^123^I]IBZM) and [^18^F]FDOPA (18% each), and [^18^F]FDG (11.5%).

Twenty-six studies were published in the last decade (2014–2024), of which 20 (76.9%) employed PET and 6 (23.1%) SPECT, using [^123^I]IBZM, [^123^I]epidepride and [^99m^Tc]ECD.

Recent years have seen the increased use of hybrid systems (Table 1), or the development of standardised methods for attenuation correction such as transmission scanning for PET-only [11, 18, 23, 30, 51–53, 58–60, 65–68, 71], and mathematical modelling for SPECT-only systems [20, 31, 41, 42, 50, 61, 62, 69]. However, earlier studies often provide limited information on attenuation correction [19, 24, 25, 27, 28, 32, 38–40, 45, 48, 49, 54–56, 64, 70], while others explicitly state it was not performed [22, 63]. One study using a PET/MRI system was included in this review and corrected for attenuation using an ultrashort echo time T1 sequence [46].

### [^18^F]FDOPA protocol

All studies required subjects to fast at least 4 h before injection, although two studies do not provide information on this [44, 46]. All studies, except one, premedicated patients with entacapone or carbidopa [46]. Most studies used activities near EANM recommendations for parkinsonism, around 150–185 MBq [9]. However, four studies administered higher doses, ranging from 318 MBq to 592 MBq [30, 36, 44, 46]. While most studies conducted a 90–95 min dynamic acquisition starting 30 s before injection, two studies followed different protocols [44, 46].

### [^18^F]FDG protocol

All studies required subjects to fast for at least 6 h, except for one study that does not provide details [43]. Either 185 or 370 MBq doses were used for each subject. In two studies, patients did a performance task for 20 min after injection [24, 25], while one study does not specify about the uptake period conditions [43]. Others indicate subjects rested for 20–40 min. Most studies do not give information on scan duration, except three which reported durations of 10, 50 and 50 min [32, 43, 45].

### Assess risk and anticipate conversion

**Fig. 4:**
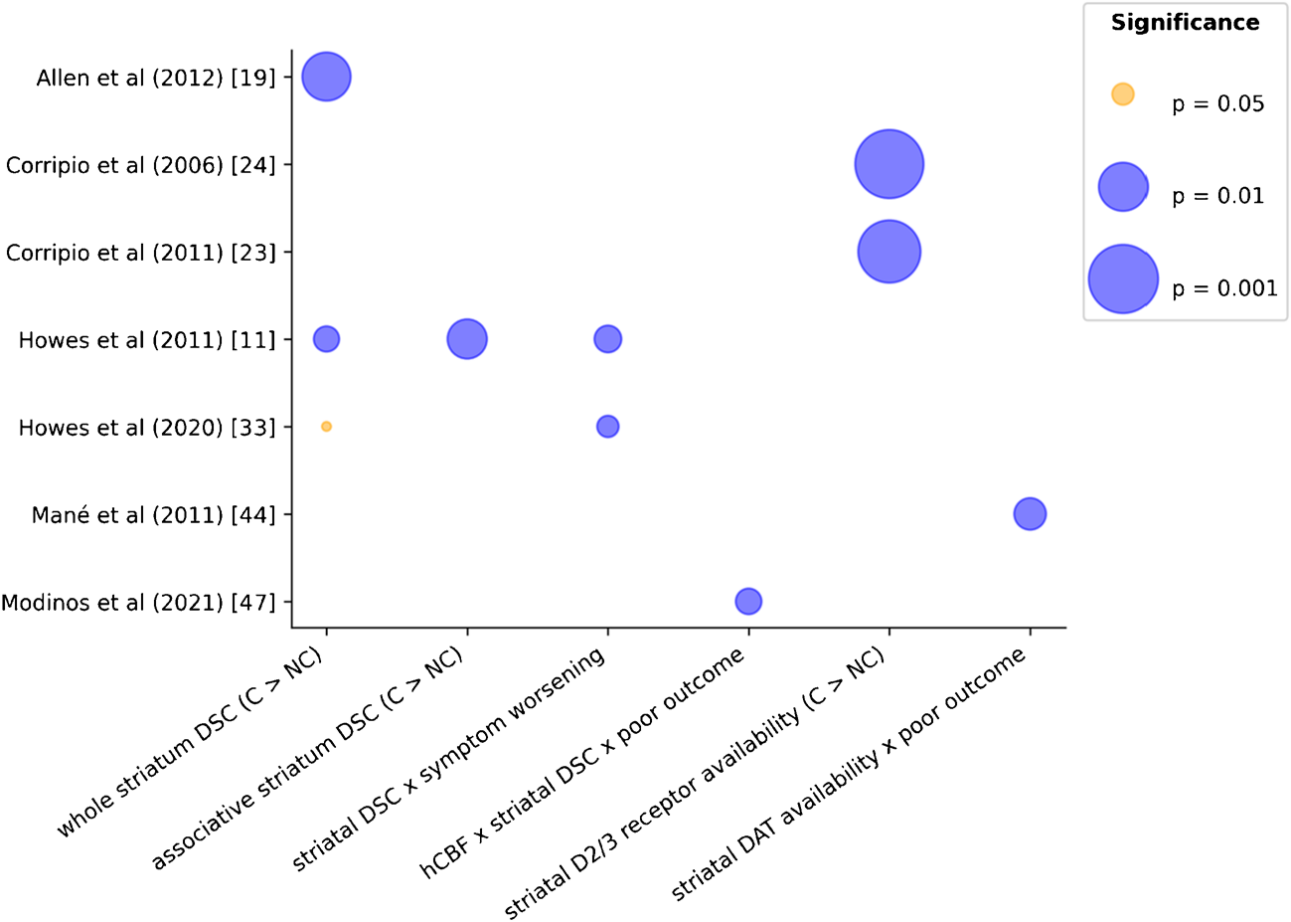
Summary of dopamine studies predicting preclinical worsening or conversion to clinical psychosis. Crosses represent direct association. Blue: significant result, yellow: non-significant result. C, converters; DAT, dopamine autotransporter; DSC, dopamine synthesis capacity; hCBF, hippocampal cerebral blood flow; NC, non-converters

The most notable finding in this category is the increased dopaminergic activity in UHR individuals who later convert to full-blown psychosis vs. those who did not. Studies used [^18^F]FDOPA, [^123^I]IBZM and [^123^I]FP-CIT to investigate presynaptic striatal dopamine synthesis, postsynaptic D2 receptor availability and presynaptic dopamine autotransporter (DAT) availability, respectively. For example, Howes et al. reports a large effect size (Cohen’s d = 1.18) in converters vs. healthy controls [11]. The bubble plot in Fig. 4 reports the different variables investigated. There was heterogeneity in the way of reporting results with sometimes no mean, confidence interval or statistic size but p-values were present in all studies.

In addition, two other studies used [^18^F]FDG and assess differences between subjects who later converted to schizophrenia vs. those who did not [24, 25]. They found significantly lower prefrontal metabolism in converters while there was no significant difference between non converters and healthy controls.

### Predict course of disease

**Fig. 5:**
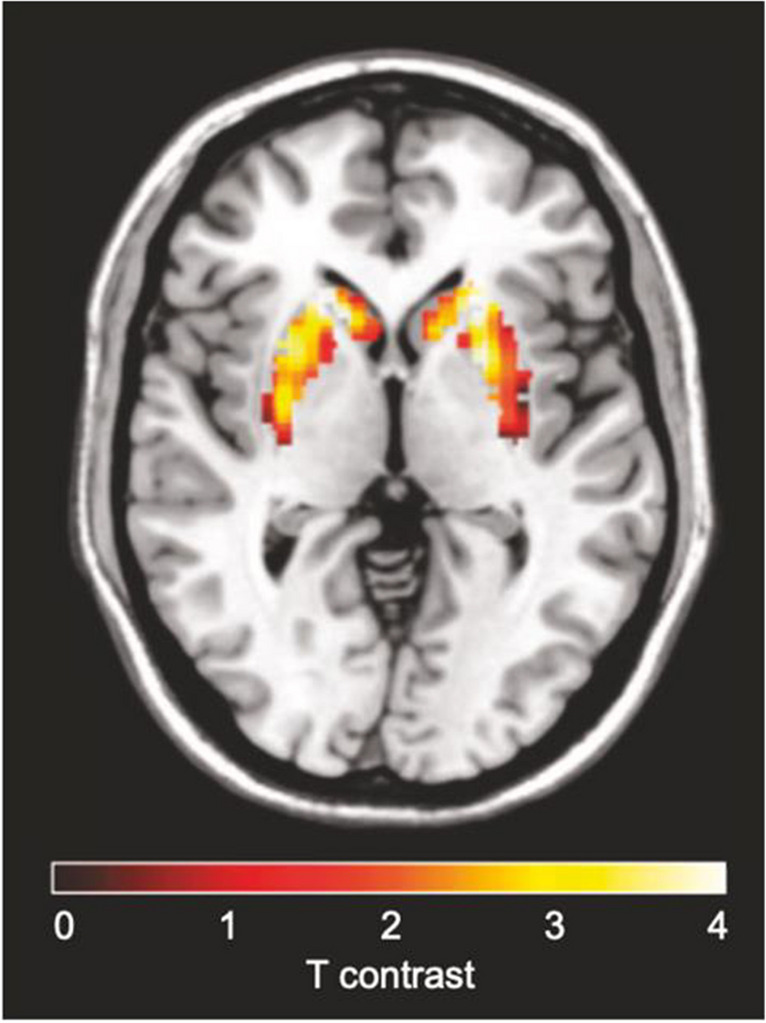
Voxel-wise analysis of responders to treatment vs. non-responders using [^18^F]FDOPA PET imaging. Adapted from Jauhar et al. [34]

Following on the subject of presynaptic dopamine hypersynthesis, Jauhar et al. carried out 3 studies measuring it in first-episode psychosis (FEP) subjects that were treatment naïve or minimally treated at the time of PET imaging [33–35]. One of their seminal findings was the relationship between increased presynaptic dopamine synthesis and response to treatment, improvement of functioning and overall remission. Effect size measured by Cohen’s d was 1.55 in responders vs. non-responders and 1.31 in responders vs. controls (Fig. 5). Their findings also suggest a prominent role of the associative striatal subdivision as well as interaction with prefrontal glutamate. These results were further supported by Sigvard et al. that demonstrated correlation between dopamine synthesis capacity and treatment response [44]. It could also have potential in predicting relapse as a negative relationship was shown between dopamine synthesis capacity and time to relapse after antipsychotic discontinuation [36]. Similarly, a study found an inverse relationship of presynaptic dopamine synthesis with negative symptoms after 3 months of treatment [46].

Regarding cerebral blood flow, studies point towards increased striatal perfusion under long-term antipsychotic treatment [28, 30, 38]. The second finding is that there may be disturbed prefrontal perfusion of other areas at baseline such as the prefrontal cortex with some signs of improvement after treatment [27, 31, 42]. Due to the interaction between perfusion and metabolism, studies on a same design using [^18^F]FDG reflect the same findings [40, 43]. More interesting for clinical translation may be studies that tried to predict symptom worsening or tardive dyskinesia using [^18^F]FDG [32, 39, 45], but these findings have not been replicated (see Table 1 for detail).

### Personalise treatment management

**Fig. 6:**
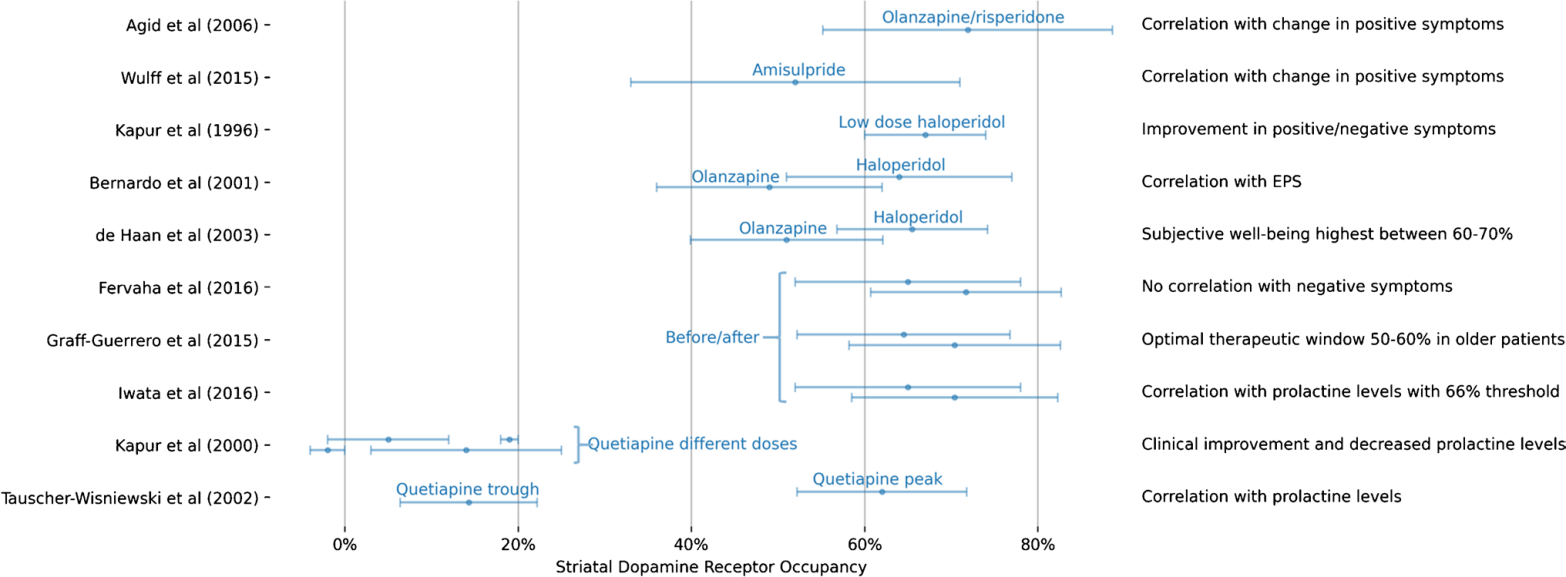
Summary of studies investigating striatal dopamine receptor occupancy under antipsychotics. Studies not reporting striatal occupancy as a percentage are not included. Markers represent mean occupancy and error bars represent one standard deviation

Most of studies that could translate to personalised medicine measure striatal post synaptic D2/3 receptor occupancy under antipsychotic treatment using [^123^I]IBZM or [^11^C]raclopride. Goals in measuring occupancy were various, but a frequent objective was correlation with symptoms or side effects (Fig. 6). Moreover, occupancy studies have shown that the relationship between medication dose and striatal dopamine receptor occupancy followed a hyperbole saturation model [55, 56, 60]. Optimal therapeutic window appears to be 65–80% in young patients and lower in older patients around 50–60% [52]. It was also shown that atypical antipsychotics such as quetiapine may only show low or transitory dopamine receptor occupancy while producing similar effects in symptoms reduction with the added benefit of reduced EPS or even reduction in prolactin levels [56]. Other studies reporting binding potentials (BP) demonstrated supportive findings. Studies investigating serotonin receptors show a similar therapeutic window for atypical antipsychotics such as quetiapine and higher binding correlates with more side effects [56, 67, 68].

## Discussion

In this systematic review, we highlight the promising use of molecular imaging at various stages of SSD. Two of the areas with the strongest evidence are measuring dopamine synthesis to predict conversion to psychosis and treatment response, as well as assessing dopamine receptor occupancy to optimise treatment. Significant heterogeneity was observed in the methods and objectives of studies, reflecting the vastness of the question asked. PET has become the preferred imaging technique over SPECT, a trend that is expected to continue. The most investigated system was postsynaptic dopamine receptors followed by presynaptic dopamine synthesis.

The onset of the first psychotic episode is often preceded by a subclinical prodrome of 1–5 years [73]. Having a biomarker to discriminate subjects most likely to convert to clinical psychosis would be of great value in allowing early disease-course modifying interventions. Longitudinal studies suggest imaging of presynaptic dopamine synthesis and postsynaptic receptor availability may be relevant and could be directed towards specific populations, such as individuals using recreational drugs [74]. Imaging of dopamine synthesis with [^18^F]FDOPA, which is already available for other indications, shows the most potential with correlation to symptom worsening replicated in several independent studies [11, 18, 21, 23]. Regarding conversion, a large effect size was identified for dopamine synthesis capacity (Cohen’s d for whole striatum = 1.18 and associative striatum = 1.24) in converters [11]. However, this was not replicated in a recent study, possibly due to a relatively short follow-up period of less than 1 year for certain patients [21].

There is also evidence to suggest that a subtype of schizophrenia is mediated through increased dopamine synthesis with correlation to positive symptoms and treatment response while the other subtype demonstrates normal dopamine synthesis and tend to not respond to typical antipsychotic treatment [6, 75]. Studies by Jauhar et al., which notably found large effect size of dopamine synthesis capacity in responders vs. non-responders (Cohen’s d = 1.55) and vs. healthy controls (Cohen’s d = 1.31), support this hypothesis [33–35]. Currently, subjects must undergo two courses of antipsychotic of adequate dose and duration to be labelled treatment-resistant. Clozapine, an atypical antipsychotic with potential life-threatening side effects such as agranulocytosis, can then be trialled and around one-third of treatment-resistant subjects will respond to this treatment [76]. A recent study evaluated a simplified 10–15 min acquisition protocol of [^18^F]FDOPA PET and assessed its economic impact if used systematically to guide treatment [77]. Despite high cost of [^18^F]FDOPA imaging, findings were in favour of a potential healthcare cost saving of ~ €3950 (~ $4250 USD) per patient due to reduced hospitalisations and faster remission. We believe that if a randomised control trial were to confirm these findings, establish a clear dopamine synthesis threshold, and present such a 10–15 min protocol suitable for clinical use instead of a 95-minute acquisition, there would be sufficient evidence to recommend [^18^F]FDOPA as an option to guide initial treatment of FEP.

Another area where there is clinical potential of molecular imaging is in personalising treatment in subjects receiving antipsychotics with high anti-dopamine receptor effect. The relationship between medication dose and clinical improvement levels off over a certain threshold resulting in diminished benefits while further side effects such as EPS, hyperprolactinaemia or abulia can be expected [78]. This has enabled to define an optimal therapeutic window around 60–80% in young subjects and lower in older subjects between 50 and 60% [52, 79]. Most studies included in our review that investigated optimisation of treatment used [^11^C]raclopride or [^123^I]IBZM to measure D2/3 receptor occupancy. Clinical translation could be facilitated using [^18^F]fallypride which has shown excellent correlation with [^11^C]raclopride and can also measure extrastriatal dopamine receptors [80]. It must be said that while these tracers allow direct assessment of receptor occupancy, indirect measure using medication plasma levels is the standard approach due to technical simplicity and cost-effectiveness [81]. However, we believe that imaging could be useful in selected cases where treatment optimisation presents challenges or when potential confounders, such as the use of substances interfering with dopamine transmission, are suspected.

Finally, the other studies presented in this review examined various systems, with a primary focus on glucose metabolism and perfusion with interchangeable results due to cerebral metabolism-perfusion coupling. Similarly to [^18^F]FDOPA, they also have the benefit of being readily available in clinical practice. Evidence suggests lower glucose metabolism in schizophrenia, particularly in chronically treated patients and a recent meta-analysis supports the theory of hypofrontality in schizophrenia [82]. It has also been observed that chronic treatment by antipsychotics could lead to hyperperfusion/hypermetabolism of striata [28, 30, 38]. Although these studies have shed light on the mechanisms of schizophrenia and treatment, it remains mostly pathophysiological at this stage and difficult to see clinical translation in the near future.

### Limitations

One limit of this work is the considerable heterogeneity of the reviewed studies at multiple levels including population (e.g. drug-naive subjects, FEP), technical aspects (e.g. choice of radiotracer, experimental design) and statistical presentation of data (e.g. size effect, voxelwise analysis). This precludes a detailed analytical perspective, such as determining an overall effect size through meta-analysis.

Despite a growing preference for PET due to its superior resolution, quantification, sensibility and rapid advancements of new technologies [83], SPECT has been employed in several recent studies, likely because of limited availability of certain radiotracers ([^11^C]raclopride, [^15^O]water). The use of SPECT introduces more variability, as seen in attenuation correction protocols while modern PET systems are typically equipped with CT or MRI. PET/MRI also enables simultaneous assessment of neurotransmitters using radionuclides and spectroscopy [84]. Further efforts are needed to develop and widely distribute reliable long half-life PET tracers.

While [^18^F]FDOPA studies generally followed standardised protocols giving further confidence into the results, [^18^F]FDG studies exhibited greater heterogeneity and sometimes omitted key information. Protocols for [^11^C]raclopride and [^123^I]IBZM imaging are not reported since, as discussed, a PET tracer such as [^18^F]fallypride would be preferable in clinical settings. However, there was also significant heterogeneity as demonstrated by the use of BP or occupancy percentage.

Other psychiatric diseases would also benefit from more standardised approaches. PET imaging suggests low levels of 5-HT1A and serotonin transporters (SERT) in major depressive disorder (MDD), possibly indicating reduced serotoninergic transmission [85–88]. This could lay the groundwork for new biomarkers in diagnosis and personalising treatment as 30–40% of MDD subjects do not respond to a first line of selective serotonin recapture inhibitor (SSRI). However, the evidence remains heatly debated, and ensuring conflicting results do not emerge from protocol inconsistency or subject heterogeneity would benefit everyone [89, 90]. Framing questions from a clinical rather than a pathophysiological perspective and further large sample size longitudinal studies would also be helpful in ensuring the emergence of imaging biomarkers in psychiatry.

### Future directions

**Fig. 7:**
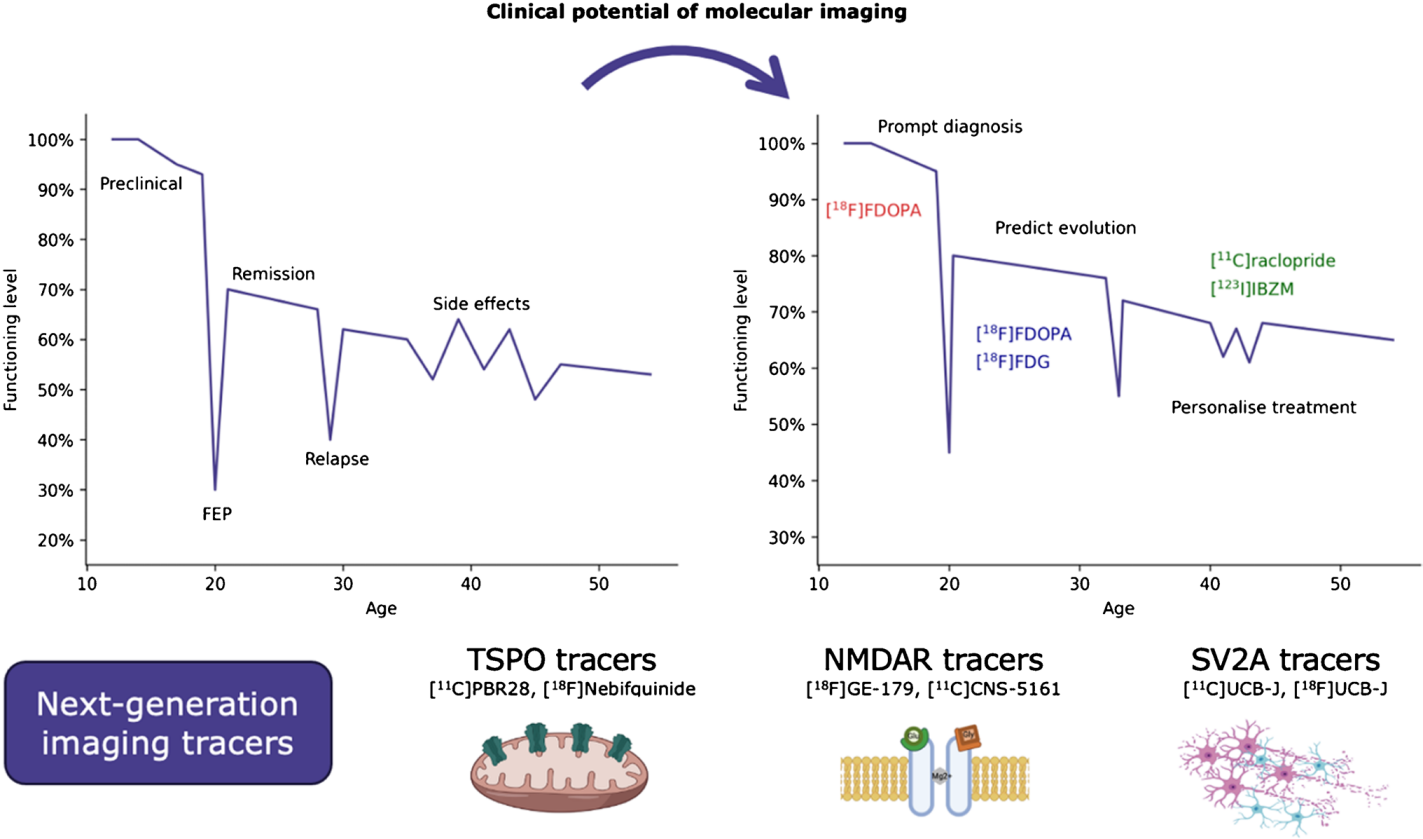
Clinical potential of molecular imaging in psychotic disorders. Dopamine and glucose imaging could lead to a change in disease course with earlier diagnosis, longer remission and fewer side effects, allowing a subject to live longer with a lower burden of disability (y-axis). Emerging techniques focus on other systems including neuroinflammation, glutamate synaptic activity and synaptic density

While many studies focus on positive symptoms, impaired functioning is also associated with the negative/cognitive aspect of the disease, which is likely mediated by other neural systems (Fig. 7). Evidence indicates that neuroinflammation may play a role in psychiatric disorders, most often studied through imaging of the mitochondrial protein TSPO found in glial cells [91]. The two longitudinal studies in this systematic review suggest different TSPO expression in schizophrenia [29, 37], and another study found correlation between symptom severity and TSPO expression in UHR subjects [92]. However, TSPO imaging is subject to severe limitations regarding its specificity and reliability due to intrinsic (i.e. biology, genetics with rs6971 polymorphism) and extrinsic (i.e. drug use, medication) factors and necessitates invasive protocols involving, for example, arterial cannulation, sedation or genotyping [93, 94].

A captivating new direction of research revolves around synaptic density [95]. It has long been known that psychotic subjects, including in the prodrome, show divergent trajectories in cortical thickness and grey matter volume compared to healthy controls likely due to excessive synaptic pruning [96, 97]. Synaptic vesicle glycoprotein 2 A (SV2A) is a ubiquitous marker of synaptic terminal density and PET radioligands, initially [^11^C]UCB-J and now labelled with fluorine-18 ([^18^F]UCB-J), have been found specific for SV2A with good correlation to synaptic density [98, 99]. Studies in patients with chronic schizophrenia found lower distribution of [^11^C]UCB-J compared to healthy volunteers in the frontal and anterior cingulate cortices with large effect sizes, and possibly lower in the hippocampus as well [100, 101]. Conversely, findings are more contradicting in the early course of schizophrenia, but therefore possibly reflecting a dynamic process in the course of disease [102, 103]. Imaging of synaptic density could become increasingly crucial, especially considering that novel drug candidates such as KarXT and Trace amine-associated receptor 1 (TAAR1) agonists do not target dopamine receptors and have been shown to enhance cognition of schizophrenic patients [104–106].

Research on ketamine, a N-methyl-D-aspartate receptor (NMDAR) antagonist used for schizophrenia modelling, and interactions between glutamate and dopamine have proved glutamatergic involvement in the pathophysiology of schizophrenia [84, 107]. There is also evidence to suggest that resistance to treatment may be underlain by high anterior cingulate glutamate levels with normal striatal dopamine synthesis [108]. Currently, imaging primarily relies on magnetic resonance spectroscopy (MRS), which measures free glutamate/glutamine level, and therefore serving as an indirect marker of NMDAR activity. Although several radiotracers have been developed to image this receptor, such as [^11^C]CNS-5161 or [^11^C]GMOM, clinical studies on schizophrenia subjects remain limited. Recent findings using [^18^F]GE-179 found lower hippocampal availability of NMDAR compared to healthy controls as well as a relationship between NMDAR availability and memory consolidation brain activity [109, 110], lending support to the NMDAR hypofunction theory.

## Conclusion

Our review highlights the potential of [^18^F]FDOPA in predicting psychosis conversion and selecting patients who could benefit from clozapine as a first-line treatment. However, a randomised clinical trial is required to confirm its impact in clinical practice. Similarly, measuring dopamine receptor occupancy could assist in personalising level of D2/3 blockade in patients on antipsychotics but necessitates further studies with long half-life PET tracers before widespread adoption. Lastly, methodological standardisation and further longitudinal studies will be paramount to enable clinical translation of innovative tracers investigating new targets such as TSPO, SV2A or NMDAR.

## Electronic supplementary material

Below is the link to the electronic supplementary material.

Supplementary Material 1

## Data Availability

No new data were generated or analysed in support of this research.
