# Supplementary material for: EANM perspective on clinical PET and SPECT imaging in schizophrenia-spectrum disorders: a systematic review of longitudinal studies
Source: Eur J Nucl Med Mol Imaging. 2024 Nov 22;52(3):876–99. doi: 10.1007/s00259-024-06987-1 (PMC11754335; doi:10.1007/s00259-024-06987-1)
Supplement: Supplementary file 1 — Supplementary Material 1 [file 259_2024_6987_MOESM1_ESM.docx]

**Supplementary materials**

**Study Protocol**

**Review question**

What can be the clinical potential of current nuclear neuroimaging techniques in schizophrenia-spectrum disorders (SSD)?

**Searches**

PubMed, Embase, Web of Science, Cochrane library will be used. The following terms are used in PubMed to identify Medical Subject Headings (MeSH) terms: "psychosis", "SSD", "schizophrenia", "PET/CT", "PET/MRI", "PET", "SPECT", "SPECT/CT", "longitudinal studies" and "follow-up". MeSH terms were extracted following the Population (psychotic disorders), Intervention (SPECT or PET imaging) and Context (longitudinal follow-up) framework. The initial search for studies ends on 14 February 2024. Any studies published after that date will not be included in this review. Unpublished studies are not sought in this review.

**Types of study to be included**

Longitudinal studies as defined by the follow-up of subjects ≥ 7 days.

**Condition or domain being studied**

Subjects diagnosed with an SSD and investigated using SPECT or PET imaging are included.

**Participants/population**

Inclusion criteria:

1. studies performed in humans with psychosis, schizophrenia or at clinical high risk of psychosis
2. using SPECT or PET imaging
3. with a longitudinal design defined as following subjects at two timepoints minimum ≥ 7 days
4. original studies, in any language

Exclusion criteria:

1. studies done in healthy human, animal or in vitro models
2. clinical trials investigating non-approved drugs
3. case reports or small series of cases (≤ 5 subjects)
4. letters to editors or commentaries
5. abstracts presented at conferences with no full text
6. phantom studies
7. reviews and meta-analyses.

**Intervention(s), exposure(s)**

Imaging using radioisotopes, i.e. PET or SPECT imaging.

**Comparator(s)/control(s)**

Depending on the studies’ design, the comparator can be high-risk individuals not converting to psychosis, non-responders to treatment or, within the same individuals, baseline symptoms/disease severity.

**Main outcome(s)**

The main outcome is to see whether PET or SPECT could predict the evolution of psychotic disorders in different contexts such as conversion to clinical psychosis in preclinical subjects, symptom worsening/improvement or response to treatment.

**Measures of effect**

Where possible, an effect size measure such as Cohen’s d is collected. If studies differ too much in the way of presenting data, and computation of measures of effect is not possible from the data reported in the articles, p-values are alternatively collected.

**Additional outcome(s)**

Not applicable

**Data extraction (selection and coding)**

Reviewer AR conduct the initial search and remove any duplication studies. Studies are imported in Rayyan software, a software dedicated to systematic reviews. Following this, reviewers AR and AJB independently use Rayyan to screen studies based on title and abstract. Both authors follow above mentioned inclusion/exclusion criteria. All articles likely to meet criteria are selected for the second step. In the second step, full-text is searched and analysed to decide upon inclusion. Researchers are blinded to each other's decisions to avoid bias. A consensus with a third author FF is searched for any disagreements. A PRISMA flow diagram is used to record the selection process. AR and FF then extracted data independently from each study including the tracer used and the system investigated (e.g. cerebral perfusion, striatal dopamine receptors etc.), the type of imaging (PET or SPECT), the number of subjects, the diagnosis, the followup period and measures of effect (see above).

**Risk of bias (quality) assessment**

Newcastle-Ottawa Scale (NOS), NIH scale for before-after studies and Cochrane Risk of Bias tool version 2 (Cochrane RoB2) are used to assess the risk of bias of the studies by 2 reviewers, AR and FF.

**Analysis of subgroups or subsets**

The review tries to explore and synthesise studies utilising the same type of PET or SPECT imaging. Specific subsets of interest will be defined after completion of data extraction and depending on the amount of literature available for different longitudinal comparisons. Plots such as forest plots are used to synthesise this type of information if useful.

**Contact details for further information**

Antoine Rogeau [arogeau.pro@gmail.com](mailto:arogeau.pro@gmail.com)

**Organisational affiliation of the review**

Department of Nuclear Medicine, Lille University Hospitals, France

Institute of Nuclear Medicine, University College London Hospitals NHS Foundation Trust, London, UK.

**Principal investigators**

Antoine Rogeau - Francesco Fraioli

**Language**

English

**Supplementary tables and figures**

**Table 1: PRISMA checklist**

**Table 2: Full search strategy Medline, Embase, the Cochrane library and Web of Science.** Boolean Operator 'AND' was used between each column.

**Table 3: Newcastle-Ottawa scale assessment for case control studies.** Exposure considered as PET/SPECT imaging. Good quality: 3 or 4 stars in selection AND 1 or 2 stars in comparability AND 2 or 3 stars in outcome/exposure. Fair: 2 stars in selection AND 1 or 2 stars in comparability AND 2 or 3 stars in outcome/exposure. Poor: 0 or 1 star in selection domain OR 0 stars in comparability domain OR 0 or 1 stars in outcome/exposure domain.

**Table 4: NIH quality assessment of before-after studies with no control group.** CD, cannot determine; N, no; NA, non applicable; NR, non reported; Y, yes. 0-33.3%: Poor; 33.3-66.7%: Fair; 66.7%-100%: Good.

**Table 5: Cochrane Risk of Bias 2 for randomised controlled study.**

**Supplementary Table 1: PRISMA checklist**

| **Section/topic** | **#** | **Checklist item** | **Location where item is reported** |
| --- | --- | --- | --- |
| **TITLE** | | |  |
| Title | 1 | Identify the report as a systematic review, meta-analysis, or both. | Title |
| **ABSTRACT** | | |  |
| Structured summary | 2 | Provide a structured summary including, as applicable: background; objectives; data sources; study eligibility criteria, participants, and interventions; study appraisal and synthesis methods; results; limitations; conclusions and implications of key findings; systematic review registration number. | Abstract |
| **INTRODUCTION** | | |  |
| Rationale | 3 | Describe the rationale for the review in the context of what is already known. | Introduction |
| Objectives | 4 | Provide an explicit statement of questions being addressed with reference to participants, interventions, comparisons, outcomes, and study design (PICOS). | Introduction - last paragraph |
| **METHODS** | | |  |
| Protocol and registration | 5 | Indicate if a review protocol exists, if and where it can be accessed (e.g., Web address), and, if available, provide registration information including registration number. | Methods - first paragraph |
| Eligibility criteria | 6 | Specify study characteristics (e.g., PICOS, length of follow-up) and report characteristics (e.g., years considered, language, publication status) used as criteria for eligibility, giving rationale. | Methods - Study selection |
| Information sources | 7 | Describe all information sources (e.g., databases with dates of coverage, contact with study authors to identify additional studies) in the search and date last searched. | Methods - Search strategy |
| Search | 8 | Present full electronic search strategy for at least one database, including any limits used, such that it could be repeated. | STable 2 |
| Study selection | 9 | State the process for selecting studies (i.e., screening, eligibility, included in systematic review, and, if applicable, included in the meta-analysis). | Methods - Study selection |
| Data collection process | 10 | Describe method of data extraction from reports (e.g., piloted forms, independently, in duplicate) and any processes for obtaining and confirming data from investigators. | Methods - Data analysis |
| Data items | 11 | List and define all variables for which data were sought (e.g., PICOS, funding sources) and any assumptions and simplifications made. | Methods - Data analysis |
| Risk of bias in individual studies | 12 | Describe methods used for assessing risk of bias of individual studies (including specification of whether this was done at the study or outcome level), and how this information is to be used in any data synthesis. | Methods - Quality assessment |
| Summary measures | 13 | State the principal summary measures (e.g., risk ratio, difference in means). | Methods - Data analysis |
| Synthesis of results | 14 | Describe the methods of handling data and combining results of studies, if done, including measures of consistency (e.g., I^2^) for each meta-analysis. | NA |
| Risk of bias across studies | 15 | Specify any assessment of risk of bias that may affect the cumulative evidence (e.g., publication bias, selective reporting within studies). | Methods - Quality assessment |
| Additional analyses | 16 | Describe methods of additional analyses (e.g., sensitivity or subgroup analyses, meta-regression), if done, indicating which were pre-specified. | NA |
| **RESULTS** | | |  |
| Study selection | 17 | Give numbers of studies screened, assessed for eligibility, and included in the review, with reasons for exclusions at each stage, ideally with a flow diagram. | Results - Eligible studies (Figure 1) |
| Study characteristics | 18 | For each study, present characteristics for which data were extracted (e.g., study size, PICOS, follow-up period) and provide the citations. | Results - Technical characteristics & STable 6 |
| Risk of bias within studies | 19 | Present data on risk of bias of each study and, if available, any outcome level assessment (see item 12). | Results - Quality assessment (Figure 2) & STables 3-5 |
| Results of individual studies | 20 | For all outcomes considered (benefits or harms), present, for each study: (a) simple summary data for each intervention group (b) effect estimates and confidence intervals, ideally with a forest plot. | Figures 4 & 5 |
| Synthesis of results | 21 | Present results of each meta-analysis done, including confidence intervals and measures of consistency. | NA |
| Risk of bias across studies | 22 | Present results of any assessment of risk of bias across studies (see Item 15). | Results - Quality assessment (Figure 2) & STables 3-5 |
| Additional analysis | 23 | Give results of additional analyses, if done (e.g., sensitivity or subgroup analyses, meta-regression [see Item 16]). | NA |
| **DISCUSSION** | | |  |
| Summary of evidence | 24 | Summarize the main findings including the strength of evidence for each main outcome; consider their relevance to key groups (e.g., healthcare providers, users, and policy makers). | Discussion paragraphs 1-5 |
| Limitations | 25 | Discuss limitations at study and outcome level (e.g., risk of bias), and at review-level (e.g., incomplete retrieval of identified research, reporting bias). | Discussion - Limits |
| Conclusions | 26 | Provide a general interpretation of the results in the context of other evidence, and implications for future research. | Discussion - Future directions of research |
| **FUNDING** | | |  |
| Funding | 27 | Describe sources of funding for the systematic review and other support (e.g., supply of data); role of funders for the systematic review. | Funding |

**Supplementary Table 2: Full search strategy PubMed, Embase, the Cochrane library and Web of Science.** Boolean Operator 'AND' was used between each column.

| **Database** | **Population** | **Intervention** | **Context** |
| --- | --- | --- | --- |
| PubMed | ("Schizophrenia Spectrum and Other Psychotic Disorders"[Mesh] OR "schizophren*"[tiab] OR "psychosis"[tiab] OR "psychotic"[tiab]) | ("Positron Emission Tomography Computed Tomography"[Mesh] OR "Positron-Emission Tomography"[Mesh] OR "Positron-Emission Tomograph*"[tiab] OR "PET/CT"[tiab] OR "PET"[tiab] OR "PET/MR*"[tiab] OR "Tomography, Emission-Computed, Single-Photon"[Mesh] OR "Single Photon Emission Computed Tomography Computed Tomography"[Mesh] OR "SPECT"[tiab] OR "SPECT/CT"[tiab]) | ("Longitudinal Studies"[Mesh] OR "Follow-Up Studies"[Mesh] OR "Prospective Studies"[Mesh] OR "followup"[tiab] OR "follow-up"[tiab] OR "longitudinal"[tiab] OR "prospective"[tiab]) |
| Embase | ('psychosis'/exp OR 'schizophrenia spectrum disorder'/exp OR 'schizophren":ab,ti OR 'psychosis':ab,ti OR 'psychotic':ab,ti) | (‘longitudinal study'/exp OR ‘follow up'/exp OR 'prospective study'/exp OR "followup":ab,ti OR "follow-up":ab,ti OR "longitudinal":ab,ti OR "prospective":ab,ti) | (‘positron emission tomography’/exp OR ‘Positron-Emission Tomography’/exp OR ‘positron-emission tomograph*’:ab,ti OR ‘PET/CT’:ab,ti OR ‘PET’:ab,ti OR ‘single photon emission computed tomography’/exp OR ‘SPECT’:ab,ti OR ‘SPECT/CT’:ab,ti) |
| Web of science | (TS=(psychosis) OR TS=(psychotic) OR TS=(schizophrenia)) | (TS=(longitudinal) OR TS=(prospective) OR TS=(followup) OR TS=(follow up) OR TS=(follow-up)) | (TS=(PET) OR TS=(PET/CT) OR TS=(PET/MR) OR TS=(positron emission tomograph*) OR TS=(SPECT) OR TS=(SPECT/CT) OR TS=(single photon emission computed tomography)) |
| Cochrane | MeSH descriptor: [Schizophrenia Spectrum and Other Psychotic Disorders] explode all trees OR ((psychosis) OR (psychotic) OR (schizophren*)):ti,ab,kw | MeSH descriptor: [Positron-Emission Tomography] explode all trees OR ((PET) OR (PET-CT) OR (PET MR) OR (positron emission tomograp*)):ti,ab,kw OR MeSH descriptor: [Tomography, Emission-Computed, Single-Photon] explode all trees OR ((SPECT) OR (SPECT-CT) OR (single photon emission computed*)):ti,ab,kw | MeSH descriptor: [Longitudinal Studies] explode all trees OR MeSH descriptor: [Prospective Studies] explode all trees OR MeSH descriptor: [Follow-Up Studies] explode all trees OR ((longitudinal) OR (prospective) OR (follow-up) OR (followup) OR (follow up)):ti,ab,kw |

**Supplementary Table 3: Newcastle-Ottawa scale assessment for case control studies.** Exposure considered as PET/SPECT imaging. Good quality: 3 or 4 stars in selection AND 1 or 2 stars in comparability AND 2 or 3 stars in outcome/exposure. Fair: 2 stars in selection AND 1 or 2 stars in comparability AND 2 or 3 stars in outcome/exposure. Poor: 0 or 1 star in selection domain OR 0 stars in comparability domain OR 0 or 1 stars in outcome/exposure domain.

| **Studies** | **Selection** | | | | **Comparability** | | **Exposure** | | |  |
| --- | --- | --- | --- | --- | --- | --- | --- | --- | --- | --- |
|  | Adequate definition | Representativeness | Selection of controls | Definition of controls | Age | Sex | Ascertainment of exposure | Same method of ascertainment | Non-Response rate - NA | Rating |
| Allen et al (2012) [19] | * |  | * | * | * | * | * | * |  | Good |
| Andersen et al (2020) [20] | * |  | * | * | * | * | * | * |  | Good |
| Brewer et al (2007) [22] | * |  |  | * | * | * | * | * |  | Fair |
| Corripio et al (2006) [24] | * |  |  |  | * | * | * | * |  | Poor |
| Corripio et al (2011) [23] | * |  |  | * | * | * | * | * |  | Fair |
| Corson et al (2002) [25] | * |  |  |  | * | * | * | * |  | Poor |
| De Picker et al (2019) [27] | * |  |  | * | * | * | * | * |  | Fair |
| Erkwoh et al (1997) [29] | * |  |  |  |  | * | * | * |  | Poor |
| Gur et al (1995) [32] | * | * | * | * | * | * | * | * |  | Good |
| Howes et al (2011) [11] | * |  | * | * | * |  | * | * |  | Good |
| Howes et al (2020) [33] | * |  | * | * | * | * | * | * |  | Good |
| Jauhar et al (2019) [36] | * |  |  | * | * | * | * | * |  | Fair |
| Jauhar et al (2019) [37] | * |  | * | * | * | * | * | * |  | Good |
| Jauhar et al (2023) [35] | * |  | * | * | * | * | * | * |  | Good |
| Kim et al (2021) [41] | * |  | * | * | * | * | * | * |  | Good |
| Laurikainen et al (2020) [42] | * |  |  | * | * |  | * | * |  | Fair |
| Livingston et al (1998) [43] | * |  |  |  | * | * | * |  |  | Poor |
| Lubeiro et al (2015) [44] | * |  |  |  | * | * | * | * |  | Poor |
| Mané et al (2011) [45] | * |  |  | * | * | * | * | * |  | Fair |
| Modinos et al (2021) [48] | * |  | * | * | * | * | * | * |  | Good |
| Molina et al (2003) [49] | * |  |  | * | * | * | * | * |  | Fair |
| Molina et al (2005) [51] | * |  |  |  | * |  | * | * |  | Poor |
| Molina et al (2005) [50] | * |  |  |  | * | * | * | * |  | Poor |
| Park et al (2019) [57] | * |  | * | * | * | * | * | * |  | Good |
| Pilowski et al (1996) [60] | * |  |  | * | * |  | * | * |  | Fair |
| Sigvard et al (2022) [66] | * |  | * | * | * | * | * | * |  | Good |
| Szymanski et al (1996) [67] | * |  |  | * | * | * | * | * |  | Fair |
| Wong et al (2022) [70] | * | * | * | * | * | * | * | * |  | Good |
| Wulff et al (2015) [72] | * |  | * | * | * | * | * | * |  | Good |
| Wulff et al (2020) [71] | * |  |  | * | * | * | * | * |  | Fair |

**Supplementary Table 4: NIH quality assessment of before-after studies with no control group.** CD, cannot determine; N, no; NA, non applicable; NR, non reported; Y, yes. 0-33.3%: Poor; 33.3-66.7%: Fair; 66.7%-100%: Good.

| **Studies** | **Q1** | **Q2** | **Q3** | **Q4** | **Q5** | **Q6** | **Q7** | **Q8** | **Q9** | **Q10** | **Q11** | **Q12** | **Rating** |
| --- | --- | --- | --- | --- | --- | --- | --- | --- | --- | --- | --- | --- | --- |
| Novak et al (2005) [56] | Y | Y | CD | NR | N | Y | Y | NR | N | N | N | NA | Fair |
| Fervaha et al (2016) [30] | Y | Y | CD | NR | Y | Y | Y | NR | Y | Y | N | NA | Fair |
| Graff-Guerrero et al (2015) [31] | Y | Y | CD | N | Y | Y | Y | NR | Y | Y | Y | NA | Good |
| Iwata et al (2016) [34] | Y | Y | Y | N | Y | Y | Y | NR | Y | Y | N | NA | Good |
| Kapur et al (1996) [38] | Y | N | CD | NR | N | Y | Y | Y | CD | N | N | NA | Fair |
| Mizrahi et al (2011) [47] | Y | Y | CD | NR | N | Y | Y | NR | CD | Y | N | NA | Fair |
| Nakajima et al (2016) [53] | Y | Y | CD | NR | Y | Y | Y | Y | Y | Y | N | NA | Good |
| Pavics et al (2004) [58] | Y | N | CD | NR | N | Y | Y | Y | CD | Y | N | NA | Fair |
| Pickar et al (1996) [59] | Y | N | CD | NR | N | Y | Y | Y | CD | Y | Y | NA | Fair |
| Rajji et al (2017) [62] | Y | Y | CD | N | Y | Y | Y | NR | Y | Y | N | NA | Fair |
| Rasmussen et al (2011) [63] | Y | N | CD | NR | N | Y | Y | NR | N | Y | N | NA | Fair |
| Rasmussen et al (2014) [64] | Y | N | CD | NR | N | Y | Y | NR | N | Y | N | NA | Fair |
| Schröder et al (1998) [65] | Y | N | CD | NR | N | Y | Y | NR | N | Y | N | NA | Fair |
| Tauscher-Wisniewski et al (2002) [68] | Y | Y | Y | N | N | N | Y | NR | Y | Y | N | NA | Fair |
| Uchida et al (2012) [69] | Y | Y | CD | NR | N | Y | Y | NR | CD | N | N | NA | Fair |

**Supplementary Table 5: Cochrane Risk of Bias 2 for randomised controlled study.**

|  | **Randomisation** | | | **Intervention** | | | | | | | **Missing data** | | | | **Measurement** | | | | | **Report** | | |  |
| --- | --- | --- | --- | --- | --- | --- | --- | --- | --- | --- | --- | --- | --- | --- | --- | --- | --- | --- | --- | --- | --- | --- | --- |
|  | **1.1** | **1.2** | **1.3** | **2.1** | **2.2** | **2.3** | **2.4** | **2.5** | **2.6** | **2.7** | **3.1** | **3.2** | **3.3** | **3.4** | **4.1** | **4.2** | **4.3** | **4.4** | **4.5** | **5.1** | **5.2** | **5.3** | **Rating** |
| Agid et al (2006) [18] | Y | PY | PN | N | N | NA | NA | NA | Y | NA | Y | NA | NA | NA | N | PN | NA | NA | NA | Y | PN | PN | Good |
|  | Low risk | | | Low risk | | | | | | | Low risk | | | | Low risk | | | | | Low risk | | |  |
| Bernardo et al (2001) [21] | Y | PY | PN | N | PN | NA | NA | NA | Y | NA | Y | NA | NA | NA | N | N | NA | NA | NA | Y | PN | PN | Good |
|  | Low risk | | | Low risk | | | | | | | Low risk | | | | Low risk | | | | | Low risk | | |  |
| de Haan et al (2003) [26] | Y | PY | N | N | PN | NA | NA | NA | Y | NA | Y | NA | NA | NA | N | N | NA | NA | NA | Y | PN | PN | Good |
|  | Low risk | | | Low risk | | | | | | | Low risk | | | | Low risk | | | | | Low risk | | |  |
| Eisenberg et al (2017) [28] | NI | Y | NI | N | N | NA | NA | NA | Y | NA | Y | NA | NA | NA | N | N | NA | NA | NA | PY | PN | PN | Fair |
|  | Concerns | | | Low risk | | | | | | | Low risk | | | | Low risk | | | | | Low risk | | |  |
| Kapur et al (1998) [40] | N | N | NI | NI | NI | NI | NI | NI | Y | NA | N | Y | NA | NA | N | N | NA | NA | NA | PY | PN | PN | Poor |
|  | High risk | | | High risk | | | | | | | Low risk | | | | Low risk | | | | | Low risk | | |  |
| Kapur et al (2000) [39] | Y | PY | PN | NI | NI | PN | NA | NA | Y | NA | N | Y | NA | NA | N | N | NI | NI | NI | PY | PN | PN | Poor |
|  | Low risk | | | Low risk | | | | | | | Low risk | | | | High risk | | | | | Low risk | | |  |
| Mishra et al (2022) [46] | Y | PY | N | Y | PY | PN | NA | NA | Y | NA | Y | NA | NA | NA | N | N | NA | NA | NA | PY | PN | PN | Good |
|  | Low risk | | | Low risk | | | | | | | Low risk | | | | Low risk | | | | | Low risk | | |  |
| Moresco et al (2004) [52] | Y | PY | PN | N | N | NA | NA | NA | Y | NA | N | N | NI | N | N | N | NA | NA | NA | PY | PN | PN | Fair |
|  | Low risk | | | Low risk | | | | | | | Concerns | | | | Low risk | | | | | Low risk | | |  |
| Nørbak-Emig et al (2016) [54] | Y | NI | N | NI | NI | NI | NI | NA | Y | NA | Y | NA | NA | NA | N | NI | NI | PN | NA | PY | PN | PN | Fair |
|  | Concerns | | | Concerns | | | | | | | Low risk | | | | Concerns | | | | | Low risk | | |  |
| Nørbak-Emig et al (2017) [55] | NI | N | N | Y | Y | NI | NI | NA | Y | NA | N | N | PN | NA | N | NI | Y | PN | NA | PY | PN | PN | Fair |
|  | Concerns | | | Concerns | | | | | | | Low risk | | | | Concerns | | | | | Low risk | | |  |
| Potkin et al (2014) [61] | Y | NI | N | Y | Y | NI | NI | NA | Y | NA | Y | NA | NA | NA | N | NI | PY | PN | NA | PY | PN | PN | Fair |
|  | Concerns | | | Concerns | | | | | | | Low risk | | | | Concerns | | | | | Low risk | | |  |
